# On the Application of Cold Spirituous Lotions to the Chest

**Published:** 1830-07-01

**Authors:** 


### COLD SPIRITUOUS LOTIONS TO THE CHEST.

In a paper signed PHILALETHES in the Medical Gazette, the writer strongly recommends the above application in certain chronic affections of an inflammatory character seated in the chest. The same has been recommended by Dr. Sutton and others in abdominal inflammation, and we have seen the suggestion for thoracic, but cannot remember the authority. The lotion which Philalethes uses is composed of seven parts of water to one of alcohol, and a little Eau de Cologne. It is applied by means of linen folded six times, across the upper part of the thorax, while the patient is in bed. It may be used tepid at first, and afterwards cold. Several cases are related in illustration, of which we shall notice two or three. The first was a young lady residing in Brompton Square, who had suffered for two years from an incessant cough. After trying several remedies Philalethes had recourse to the spirit lotion. From that day the cough abated, and in a month ceased in toto. The next case was that of Captain M. of the India Merchant Service, who had had a troublesome cough for months, with pain in the cardiac region. Other remedies failing, the lotion was tried, and, in a week or two, with decided benefit. In a month he was well.

Mr. Smith, a clergyman in Cambridge, came to the author of the paper with pain in his side, and "many symptoms threatening phthisis." He thought the case full of danger, if not of despair. He recommended the cold lotion, the mildest kinds of animal food, gentle exercise. He recruited, lost his cough, and ultimately the pain in his side. Philalethes thinks, however, there are tubercles. He does not propose the lotion as a remedy for phthisis; but as more or less of pleuritis frequently attends tubercular consumption, he thinks the lotion will be beneficial in that case. He has applied the alcoholic lotion in hæmoptysis, and with, he conceives, great advantage. In a word, "he would recommend the spirit lotion in most, or all, chronic cases of pectoral affection. In many we shall have the pleasure of seeing the patient recover under its use—in none can it do injury." He has never known it give cold; but he generally takes the precaution of applying it tepid at first, and causing it to be used under the bed-clothes at night.

It would have been more satisfactory if Philalethes had given a more distinct estimate of the actual condition of the thoracic organs, as ascertained by means which have now happily triumphed over all opposition. We hear no more of sarcasms against auscultation and percussion. Those who first opposed the stethoscope openly, as a bauble, a piece of quackery, &c. have either availed themselves of the advantages resulting from its application, or become prudently silent on the opposition benches.

